# Downregulation of serum RAB27B confers improved prognosis and is associated with hepatocellular carcinoma progression through PI3K-AKT-P21 signaling

**DOI:** 10.18632/oncotarget.18010

**Published:** 2017-05-19

**Authors:** Xue Yang, Xieqiong Ye, Le Sun, Fangyuan Gao, Yuxin Li, Xiaomin Ji, Xuejiang Wang, Ying Feng, Xianbo Wang

**Affiliations:** ^1^ Center of Integrative Medicine, Beijing Ditan Hospital, Capital Medical University, Beijing, China; ^2^ Dongzhimen Hospital, Beijing University of Chinese Medicine, Beijing, China; ^3^ Department of Physiology and Pathophysiology, School of Basic Medical Sciences, Capital Medical University, Beijing, China; ^4^ Beijing Key Laboratory for Cancer Invasion and Metastasis Research, Capital Medical University, Beijing, China

**Keywords:** RAB27B, PI3K/AKT, p21, cell proliferation, hepatocellular carcinoma

## Abstract

Previous study revealed that elevated expression of RAB27B in tissues is correlated with hepatocellular carcinoma (HCC) progression; however, the mechanisms involved in promoting HCC development are still unclear. Moreover, HCC tissues are not readily obtained during routine diagnosis. Therefore, to further explore its potential value in early diagnosis, we examined RAB27B expression in patient sera. First, the correlation between serum RAB27B expression and survival, as well as TNM and Barcelona Clinic Liver Cancer stages, were evaluated in patients with HCC. Second, lentiviral vector plasmids carrying interference sequences and plasmids harboring the complete open reading frame of RAB27B were designed to knockdown or overexpress RAB27B in BEL7402 or HuH-7 cells to determine its biological function. Compared with healthy controls and patients with chronic hepatitis B infection, serum RAB27B was significantly increased in patients with HCC. After down-regulating expression of RAB27B, the proliferation of BEL7402 cells was remarkably inhibited both *in vitro* and *in vivo*. Additionally, activation of the PI3K/AKT pathway was significantly diminished. Moreover, cell cycle progression of the knockdown cells was notably arrested in the G1/S phase, and upregulation of p21 contributed to this effect. Restoration experiments to recover RAB27B expression revealed opposing results. These findings indicated RAB27B might regulate cell cycle through the PI3K/AKT/p21 pathway by releasing cytokines via exocytosis, thereby modulating the proliferation of HCC cells. RAB27B could potentially be a valuable serum biomarker for the early diagnosis of, and a therapeutic target in, HCC.

## INTRODUCTION

Primary liver cancer (PLC) is the sixth-most common malignant cancer and the third-ranked cause of cancer-related deaths, with a high rate of malignancy and mortality worldwide [1]. Hepatocellular carcinoma (HCC), accounting for more than 85% of PLC, is the main tissue subtype in PLC [2, 3]. Despite advances in therapeutic modalities for HCCs, the 5 year survival rate remains low compared with that in other types of cancers [4]. The lack of obvious signs and symptoms in the initial stage of HCC has resulted in most patients missing early treatments. Accordingly, exploring the mechanisms involved in the occurrence and progression of HCC and determining potential prognostic targets is of great clinical significance.

As with other tumors, the occurrence and progression of HCC is closely related to dysregulation of the cell cycle, which can lead to excessive and uncontrolled cell proliferation. The cell cycle is regulated by the activity of cyclin-dependent kinases (CDKs) and cyclin-dependent kinase inhibitors (CDKIs), including p21 [5, 6]. The activity of p21 is governed by mitotic cascade signaling and cell damage responses, and studies have shown that its function is related to its subcellular location [7–10]. Studies have revealed that the activation of AKT enables phosphorylation of two residues of p21, T145 and T146. Phosphorylation at T145 induces the translocation of p21 from the nucleus to the cytoplasm, where it exerts its pro-cell growth and anti-apoptotic effects [11–13].

Vesicle transport plays an important role in the exchange of substances and signals inside and outside the cell [14, 15]. Rab GTPases belong to a family of conserved transport proteins that regulate vesicular transport in all eukaryotes [16]. RAB27B, a member of the Rab27 subfamily, participates in the processes of exocytosis. Other than in brain tissues, expression of RAB27B is relatively low in normal tissues [17]. Previous studies have found that increased expression of RAB27B is related to malignant progression of cancers, including breast [18], [19], HCC [20], ovarian cancer [21], and glioblastomas [22]. Recent research has revealed that elevated expression of RAB27B is correlated with the progression of HCC; however, the role of RAB27B in promoting the progression of HCC remains unclear [20].

Based on the above findings, this study evaluated the underlying mechanism and possible signaling pathways adopted by RAB27B in the progression of HCC. By knocking down or increasing RAB27B expression separately in BEL7402 and HuH-7 cells, we evaluated the function of RAB27B in promoting cell proliferation *in vitro* and *in vivo*. Downregulation of RAB27B inhibited the activation of PI3K and AKT, stabilizing p21 in the nucleus where it exerts an inhibitory effect on the cell cycle.

Moreover, in this study, we assessed the differential expression of RAB27B in the sera of patients with HCC, as well as in that of normal humans and patients with chronic hepatitis B infection, to elucidate its potential significance in early diagnosis of HCC.

In conclusion, this study demonstrated that RAB27B had the ability to promote cell proliferation by regulating the PI3K/AKT/P21 pathway and provided an augmented ease of diagnosis and the identification of new candidate targets for HCC therapy.

## RESULTS

### High serum RAB27B expression was associated with poor prognosis in patients with HCC

**Figure 1:**
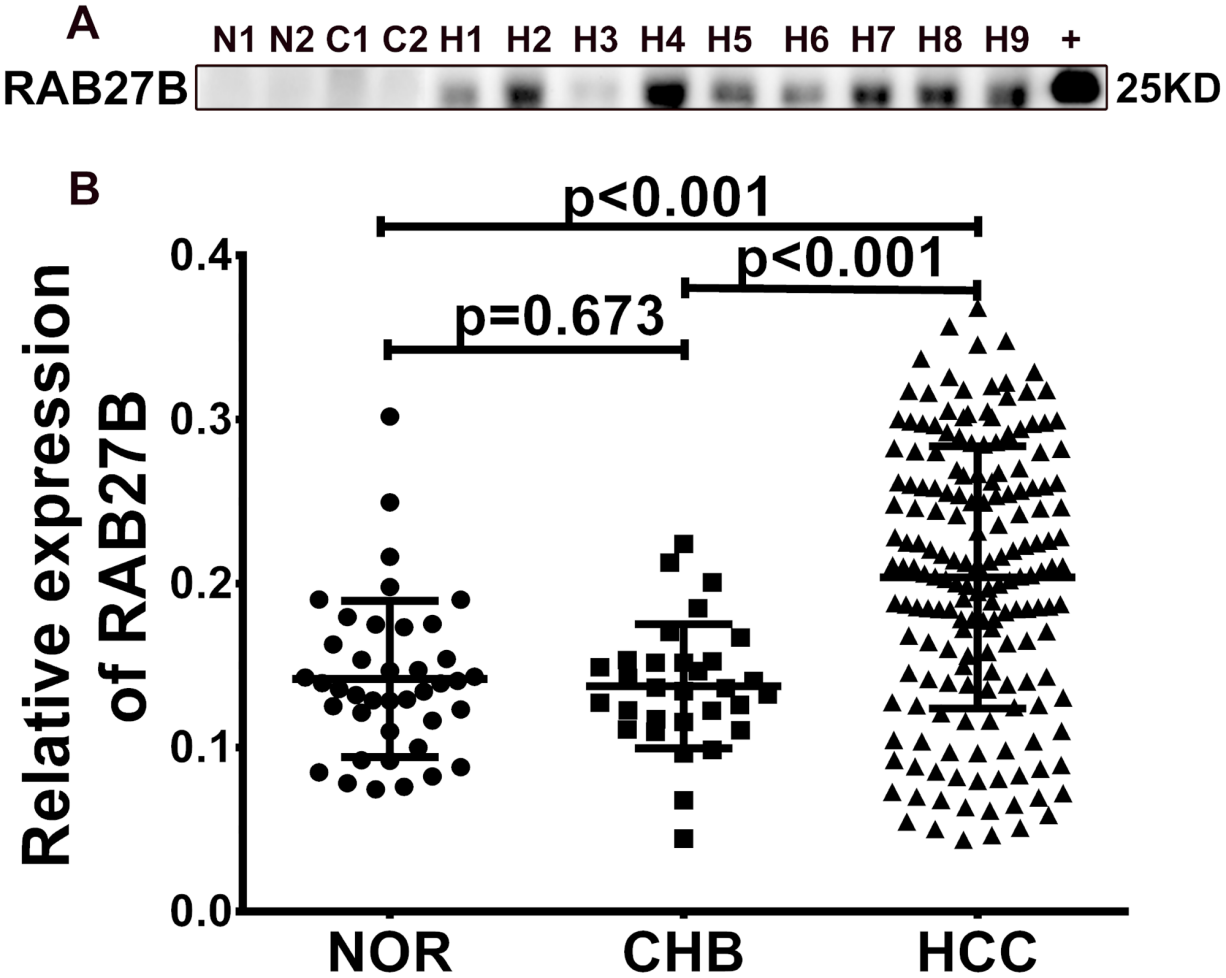
RAB27B is highly expressed in the serum of patients with hepatocellular carcinoma (HCC) **(A)** Serum RAB27B expression level was determined by western blotting. RAB27B protein was used for the positive control as well as semi-quantitative index. **(B)** With the gray value of RAB27B protein as the standard, the gray value and concentration of serum RAB27B was statistically calculated and analyzed. Compared with NOR and CHB patients, the expression of RAB27B was significantly higher in patients with HCC (*p* < 0.001, *p*<0.001). There was no difference between NOR and CHB patients (*p* = 0.673). N = NOR = normal individuals, C = CHB= chronic hepatitis B, H = HCC = hepatocellular carcinoma, +=RAB27B fusion protein.

Western blotting was carried out to evaluate the expression of RAB27B in serum samples from 154 patients with HCC, 40 healthy controls, and 31 patients with chronic hepatitis B infection (Figure 1A, Supplementary Figure 1 and 2A). Compared with that from normal humans and patients with chronic hepatitis B infection, RAB27B in sera from patients with HCC exhibited significantly increased levels (Figure 1B, *p* = 0.673, *p* < 0.001, *p* < 0.001).

A total of 154 patients with HCC were included in our study. Baseline characteristics of the study population are presented in Table 1. To determine the predictive factors for the survival of HCC, the prognostic values of the variables were evaluated.

**Table 1:** Univariate and multivariate analysis of OS in patients with HCC (N=154)

Variable	HCC^#^	Univariate analysis		Multivariate analysis	
		HR(95% CI)	*P*-value	HR(95% CI)	*P*-value
Men/women	133 / 21	0.660(0.230, 1.860)	0.433		
Age,y	53.5±7.4	0.960(0.920, 1.000)	0.092		
Hepatic encephalopathy	7	3.160(1.190, 8.370)	0.020		
Ascites	57	3.520(2.330,5.330)	<0.001		
Number of tumors ≥3	83	5.560(2.330,13.330)	<0.001		
Tumor size≥5 cm	32	6.520(3.430,12.440)	<0.001		
Lymph node metastasis	17	3.060(1.450,6.490)	0.003		
Portal vein involvement	34	7.830(4.050,11.130)	<0.001		
ALT(IU/L)	30.4(22.0-43.3)	1.006(1.002,1.010)	0.007		
AST(IU/L)	68.2±178.8	1.001(1.001,1.002)	<0.001		
TBiL(μmoI/L)	19.1(11.8,33.4)	1.010(1.006,1.013)	<0.001		
ALB(g/L)	37.1±6.6	0.950(0.917,0.988)	0.010		
ALP(IU/L)	86.9(66.8, 125.1)	1.001(1.001,1.002)	<0.001		
GGT(IU/L)	46.2(30.2,98.9)	1.006(1.004,1.007)	<0.001		
PTA (%)	78.3±16.8	0.980(0.960,1.000)	0.067		
NLR	2.4(1.7,3.9)	1.130(1.070,1.190)	<0.001		
Cr(μmoI/L)	65(57,75.2)	1.0(0.990,1,020)	0.497		
AFP	5.1(2.4,95.1)	1.0(1.0,1.0)	<0.001		
HBV DNA≥ 500 IU/ml	52	7.200(3.480,14.860)	<0.001		
Child-Pugh score	6(5,7)	1.530(1.330,1.770)	<0.001		
BCLC stages (A/B/C/D)	62/45/29/8	2(2, 5)	<0.001		
TNM grades (I/II/III/IV)	53/53/32/16	1(0,2)	0.275		
RAB27B	205.4±78.5	1.007(1.003,1.012)	0.002	1.007(1.001,1.013)	0.022

AFP: a-fetoprotein; ALB: serum albumin; ALP: alkaline phosphatase; ALT: alanine aminotransferase; AST: aspartate aminotransferase; Cr: serum creatinine; GGT: g-glutamyltranspeptidase; NLR: neutrophil–lymphocyte ratio; PTA: prothrombin activity; TBil: total bilirubin; CI: confidence interval; HR: hazard ratio; OS: overall survival; HCC: hepatocellular carcinoma

^#^Data are presented as the number of observations or the mean±standard deviation.

Univariate analysis showed that RAB27B, ALT, AST, TBil, ALB, ALP, GGT, NLR, AFP, tumor number ≥ 3, tumor size ≥ 5 cm, HBV DNA ≥ 500 IU/ml, Child-Pugh score, hepatic encephalopathy, ascites, lymph node metastasis, BCLC stages and portal vein involvement were associated with decreased overall survival in patients with HCC (*p* < 0.05, Table 1). By multivariate Cox regression analysis, only RAB27B was significantly associated with overall survival time (OST) (*p* < 0.05, Table 1).

**Figure 2:**
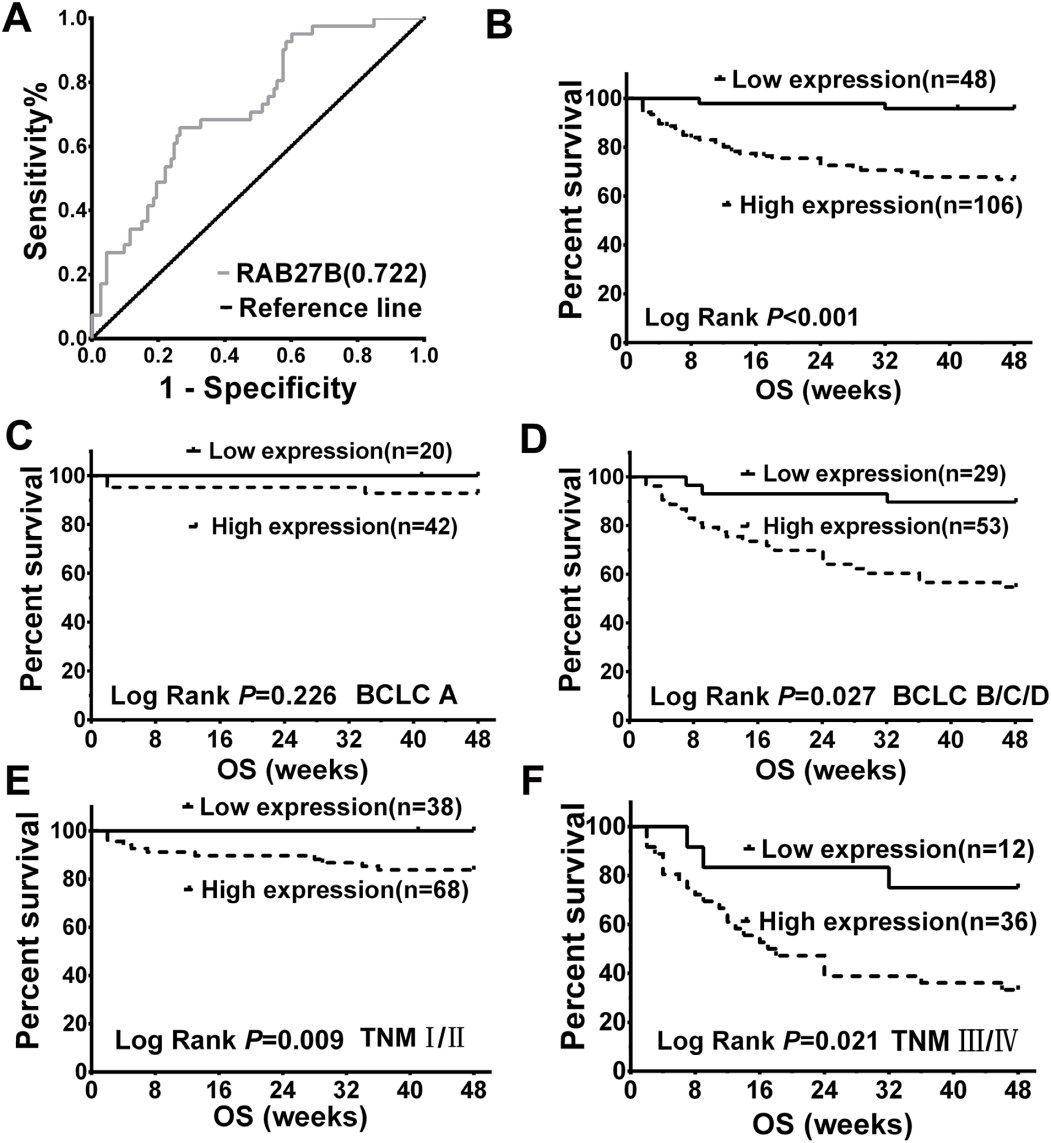
Serum expression of RAB27B in patients with hepatocellular carcinoma (HCC) was related to overall survival time and malignancy **(A)** ROC curves revealed association between serum RAB27B expression and OST by multivariate analysis in patients with HCC. The AUC was calculated to evaluate the discriminatory capacity of RAB27B in predicting the survival of patients with HCC (AUC = 0.722, cutoff value = 0.182, *p* < 0.05). **(B)** According to the cutoff value, patients were divided into two groups: high expression (n = 106, serum RAB27B ≥ 0.182 fg/ml) and low expression (n = 48, serum RAB27B < 0.182 fg/ml). Associated with patients’ OST, K-M analysis was carried out between the two groups. The results showed that patients with higher expression of RAB27B may have a shorter OST (*p* < 0.001). **(C, D)** Based on the cutoff value and BCLC stages, patients in BCLC stage A and stages B/C/D were divided into two groups: BCLC-A low expression (n = 20, serum RAB27B < 0.182 fg/ml) and high expression (n = 42, serum RAB27B>=0.182 fg/ml) groups; BCLC-B/C/D high expression (n = 53, serum RAB27B ≥ 0.182 fg/ml) and low expression (n = 29, serum RAB27B < 0.182 fg/ml) groups. Results revealed that patients in BCLC B/C/D groups who had a lower expression of RAB27B might have a longer OST (*p* = 0.027) **(E, F)** According to the cutoff value and TNM grades, patients in TNM-I, II, TNM-III, IV were separately divided into two groups: TNM-high expression (n = 68, serum RAB27B < 0.182 fg/ml) and low expression (n = 38, serum RAB27B ≥ 0.182 fg/ml) groups; TNM-III, IV high expression (n = 36, serum RAB27B ≥ 0.182 fg/ml) and low expression (n = 12, serum RAB27B < 0.182 fg/ml) groups. Higher expression of serum RAB27B was correlated with shorter OST both in TNM I-II and III-IVgrades (*p* = 0.009, *p* = 0.021). OST: overall survival; ROC: receiver operating characteristic; AUC: area under curve; K-M analysis: Kaplan-Meier analysis.

To analyze the value of quantification of serum RAB27B in the prognosis of HCC, we prepared the receiver operating characteristic (ROC) curve for serum RAB27B expression according to OST. The area under the ROC curve (AUC) value for RAB27B was 0.722, and the cut-off value was 182 fg/ml (*p* < 0.05, Figure 2A). These results suggest that serum RAB27B level in patients with HCC has potential diagnostic value. Patients with HCC were divided into high- and low-expression groups according to the cutoff value. Kaplan-Meier (K-M) survival analysis revealed that patients with lower expression of RAB27B had significantly longer OST (*p* < 0.001, Figure 2B).

RAB27B expression was also analyzed according to the BCLC stages of patients with HCC. Results revealed that patients with higher serum levels of RAB27B had a significantly poorer prognosis at BCLC stage BCD (*p*= 0.027, Figure 2D). For patients in BCLC stage A, the correlation between RAB27B expression and prognosis was not prominent (*p* = 0.226, Figure 2C). Moreover, analysis was carried out based on the RAB27B expression and OSTs of patients with HCC according to patients’ TNM grades. K-M survival analysis revealed that patients with higher expression of RAB27B tended to have a poorer prognosis both in TNM I-IIand III-IV grades (*p* = 0.09, *p* = 0.021, Figure 2E and 2F).

### Inhibition of cell proliferation and cell cycle progression in RAB27B-knockdown BEL7402 cells

**Figure 3:**
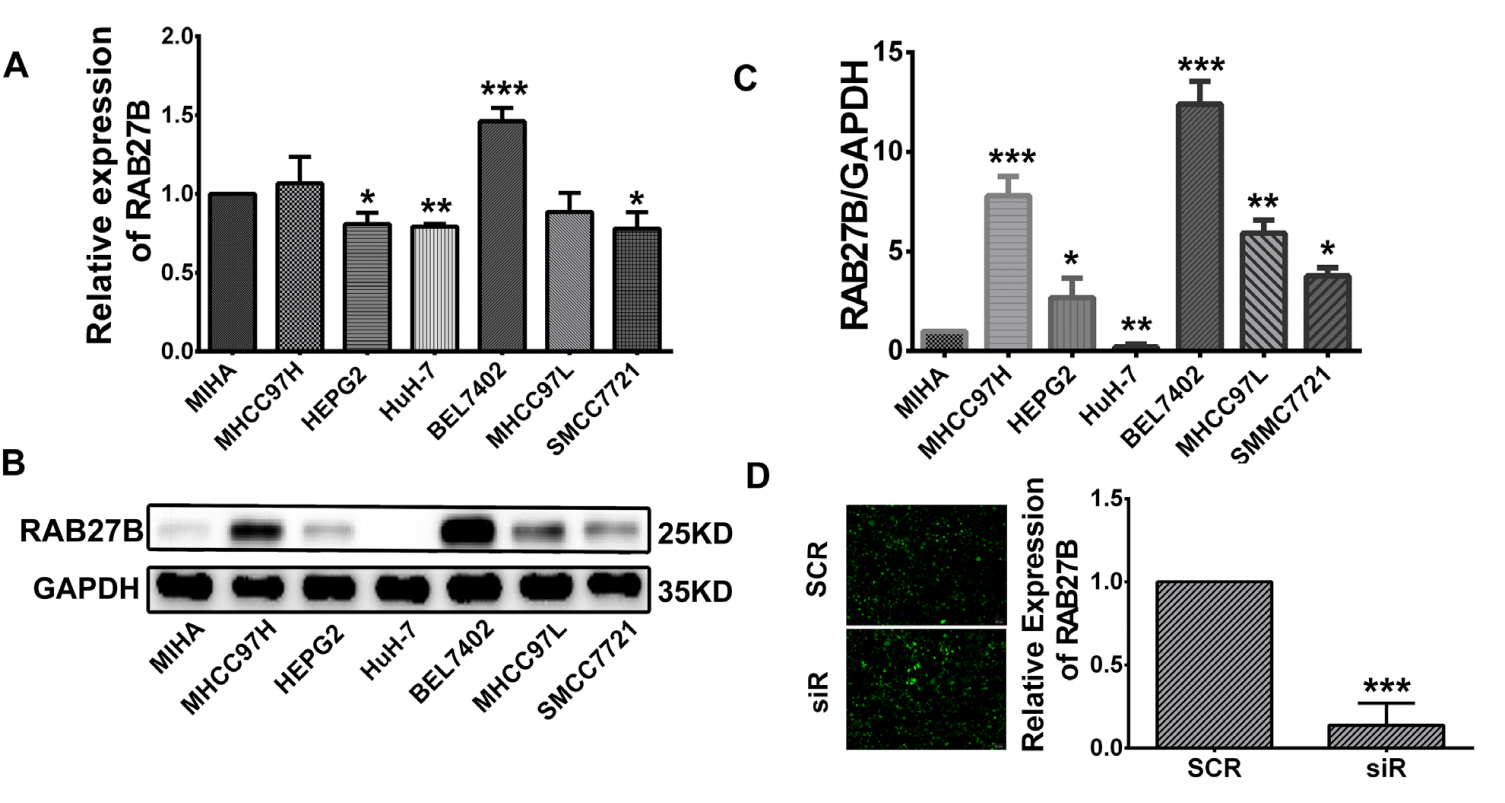
Cell infection **(A, B, C)** RAB27B expression in HCC cell lines (MHCC97H, HEPG2, HuH-7, BEL7402, MHCC97L, and SMCC7721) was compared with that in a normal liver cell line (MIHA) by western blotting and RT-qPCR. **(D)** Fluorescence microscopy was used to observe the transfection efficiency of GFP-plasmid 72 hours after infection. The infection efficiency was above 80%. RT-qPCR analysis was carried out to evaluate the knockdown efficiency of the GFP-plasmid 72 hours after infection. Knockdown efficiency was greater than 80%. *p < 0.05, **p < 0.01, ***p < 0.001.

Expression of RAB27B was detected in 7 common HCC cell lines, including BEL7402, MHCC97H, MHCC97L, HuH-7, SMMC, and HEPG2, as well as in the normal liver cell line, MIHA. Western blot and RT-qPCR results showed that RAB27B was highly expressed in metastatic cell lines, especially in BEL7402 cells. HuH-7 cells expressed a low level of RAB27B, at both the mRNA and protein levels (Figure 3A, 3B, 3C). BEL7402 cells are an AFP-positive, metastatic cell line, and have the highest level of RAB27B expression. Hence, we selected it to detect the function of RAB27B in this study. SiRNA-RAB27B and scramble plasmids, named as siR, and SCR, were constructed with GFP-lentivirus plasmids. The siR plasmid was used to knockdown the expression of RAB27B to further determine its biological role in tumor cell proliferation and cell cycle (Figure 3D). As revealed by RT-qPCR, the level of RAB27B was significantly downregulated after transfection with lenti-siR (*p* < 0.001, Figure 3D).

**Figure 4:**
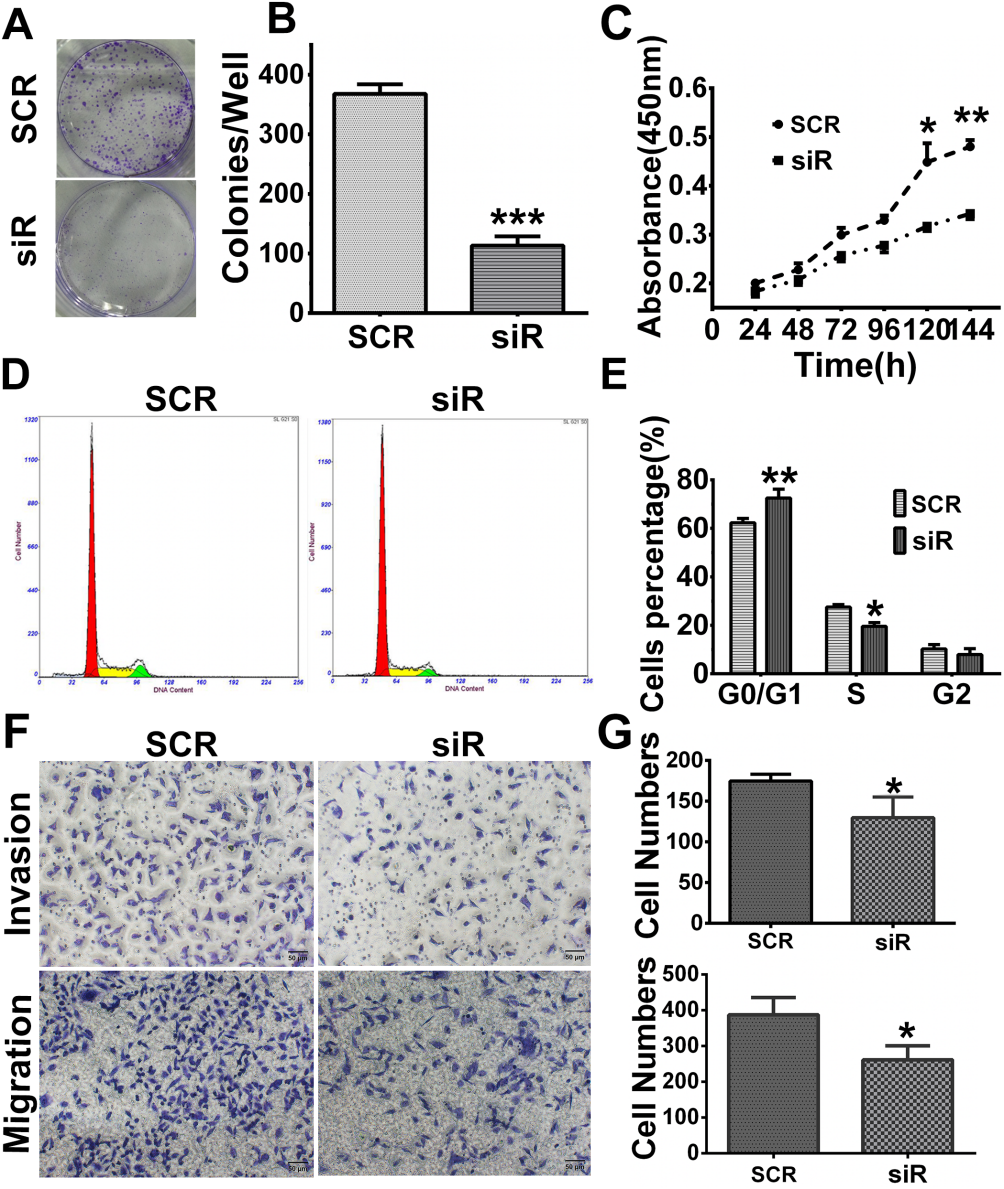
Knockdown of RAB27B inhibits cell proliferation, migration, invasion and G1/S transition in BEL7402 cells **(A, B)** Cell colony formation ability was detected by colony formation assay. **(C)** The MTT assay showed a remarkable arrest after knockdown for 120 hours. **(D, E)** Flow cytometry analysis revealed cells transfected with the GFP- plasmid- siRNA-RAB27B (siR) for 72 hours were remarkably halted at the G0/G1 stage. Cells in S stage were significantly decreased. **(F, G)** Cell migration and invasion assays were analyzed in BEL7402 cells infected with siR or scramble sequence (SCR). *p < 0.05, **p < 0.01, ***p < 0.001.

Cell proliferation was analyzed by MTT and colony formation assays. After infection with lenti-SCR/siR plasmids for 24 hours, cells were seeded in 96-well plates. The results of the colony formation assay showed that cells which expressed a lower level of RAB27B displayed smaller and fewer colonies than cells transfected with the SCR plasmid (*p* < 0.001, Figure 4A and 4B). As revealed by the MTT assay, cell growth was remarkably inhibited by downregulation of RAB27B compared with SCR cells after 120 hours (*p* < 0.05, Figure 4C). These results indicate that the natural existence RAB27B could constantly promote HCC cell growth. In the regulation of cell cycle, after knockdown of RAB27B by siR plasmids for 72 hours, the number of cells in the G0/G1 stage was significantly increased, whereas there was a dramatic decrease in the number of cells in the S stage. Knock-down of RAB27B lead to significant cell cycle arrest (*p* < 0.01, *p* < 0.05, Figure 4D and 4E). Cell migration and invasive abilities were also analyzed after infecting siR/SCR plasmids for 24 hours. The results identified that cell migration and invasion were greatly restrained after down-regulation of RAB27B expression (*p* < 0.05, *p* < 0.05, Figure 4F and 4G). Therefore, we suspect that RAB27B may promote cell proliferation and cell cycle progression in HCC cells.

### Recovery experiments

**Figure 5:**
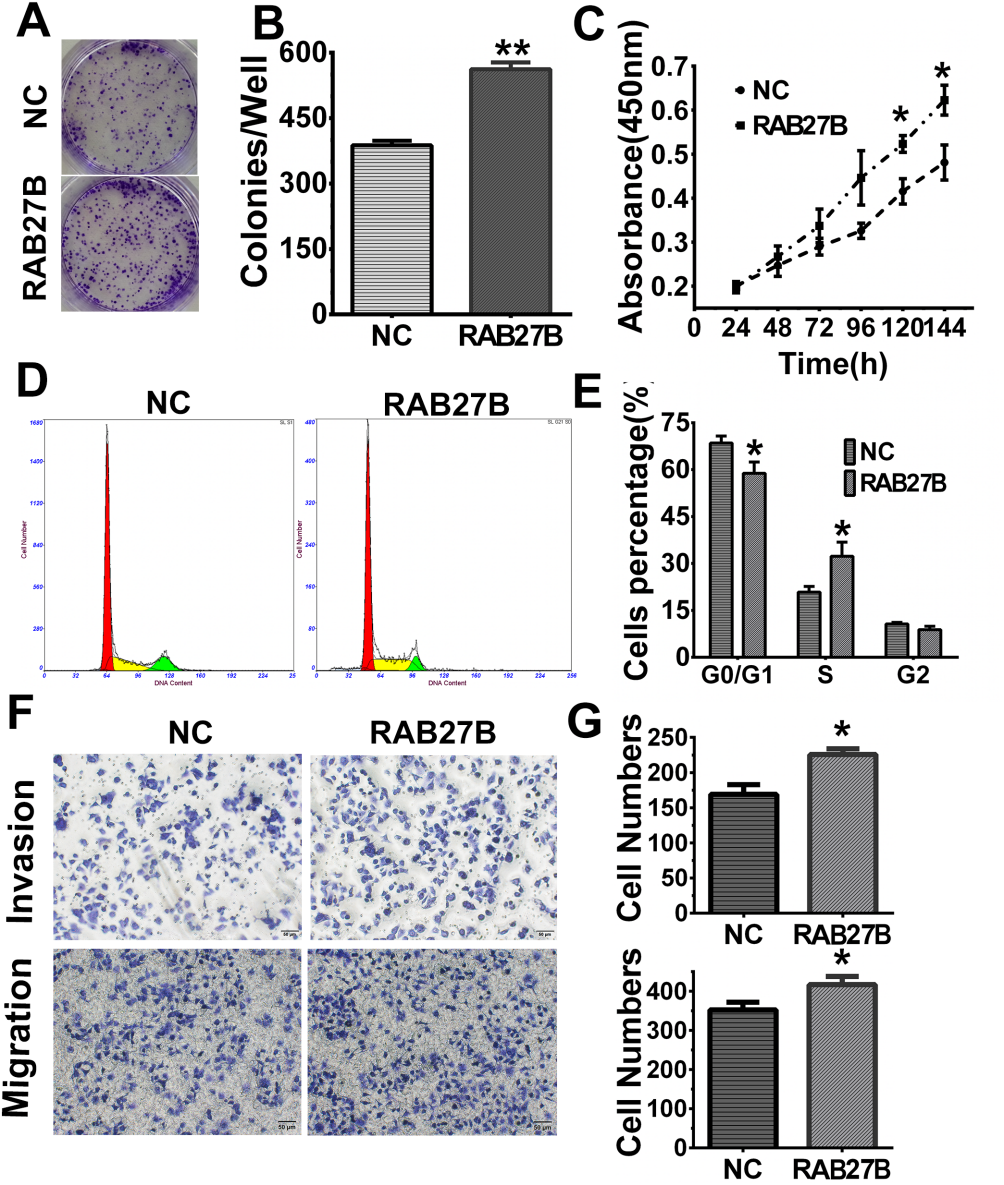
Upregulation of RAB27B promotes proliferation, migration, invasion and G1/S transition in HuH-7 cells **(A, B)** After transfection with Negative Control (NC)/RAB27B plasmids for 24 h, HuH-7 colony formation ability was increased remarkably, and **(C)** in MTT assays, cell proliferation was significantly improved after transfection for 120 hours. **(D, E)** Cell cycle analysis also revealed that the number of cells in S phase was greatly increased in RAB27B-transfected cells compared to negative control (NC) cells, whereas cells in G0/G1 stages were greatly decreased. **(F, G)** After transfection with NC or RAB27B plasmids, cell migration and invasion ability were detected in HUH7 cells. *p < 0.05, **p < 0.01, ***p < 0.001.

To verify the knockdown experimental results, we designed a recovery experiment to discern the biological functions of RAB27B in HuH-7 cell lines with relatively low endogenous expression levels of the protein. After transfection of RAB27B plasmids for 24 h, cells showing higher expression levels of RAB27B displayed greater numbers and physically larger colonies than Negative Control (NC)-transfected cells (*p* < 0.01, Figure 5A and 5B). The MTT results indicated that cell proliferation of RAB27B -transfected cells was significantly elevated after 96 h (*p* < 0.05, Figure 5C). These findings revealed that exogenous RAB27B enhanced HCC cell proliferation over time. Cell cycle analysis yielded comparable results. There was a much greater number of cells in S phase after transfection of RAB27B for 72 hours, whereas there was a remarkable decrease in the number of cells in the G0/G1 stages (*p* < 0.05, *p*< 0.05, Figure 5D and 5E). After transfection with RAB27B/NC plasmids for 24 h, cell migration and invasion abilities were detected and these indices were significantly increased (*p* < 0.05, *p* < 0.05, Figure 5F and 5G).

### RAB27B promotes HCC cell growth by activating the PI3K/AKT pathway and down-regulating p21

**Figure 6:**
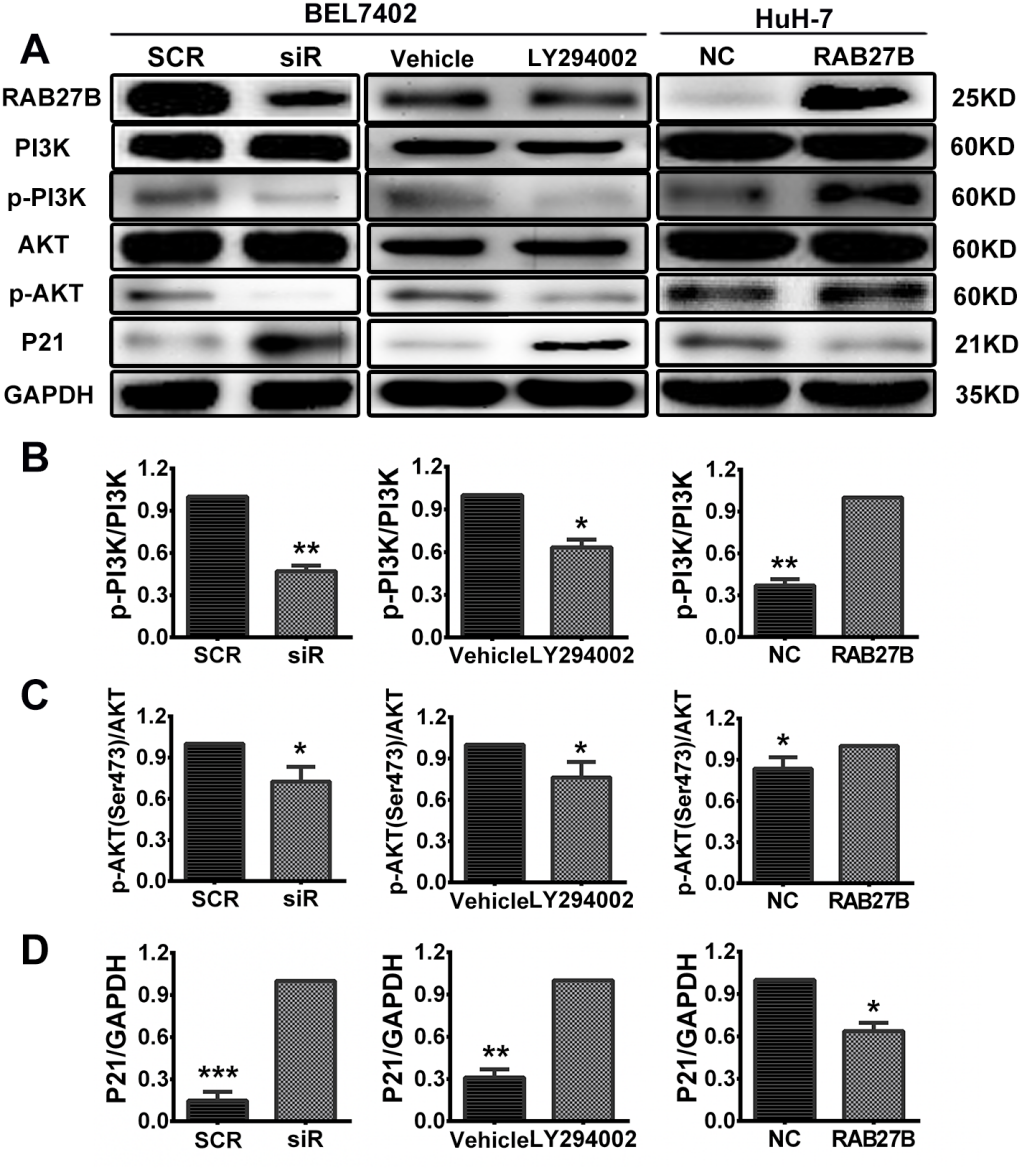
Knockdown of RAB27B inhibits the PI3K/AKT pathway and stabilizes p21^waf/Cip1^ to suppress HCC cell proliferation **(A)** Expression of proteins participating in the PI3K/AKT/p21 pathway was evaluated. **(B, C, D)** The gray value statistics of protein p-PI3K, p-AKT and p21. *p < 0.05, **p < 0.01, ***p < 0.001.

Vesicle transport plays a key role in the transportation of materials and signals across cell membranes. RAB27B plays a role in the process of exocytosis, transporting proteins and growth factors into the extracellular milieu. In our study, we identified that downregulating RAB27B for 72 to 96 hours could remarkably inhibit the PI3K/AKT pathway through the transportation of cytokines. Expression of p-PI3K and p-AKT (Ser473) were downregulated with the decreased expression of RAB27B (Figure 6A). These results indicate that RAB27B can regulate cell proliferation through the PI3K/AKT pathway.

During the cell cycle, CDKs and CDKIs contribute to the transition between phases. Among these proteins, p21, a CDKI, plays a key role in the transition of the cell cycle between G1 and S phases. Furthermore, previous research has confirmed that the stability of p21 is controlled by the PI3K/AKT pathway. P-AKT could promote the transition of p21 from the cell nucleus to the cytoplasm, where it can then exert its function in promoting cell proliferation. As the results above revealed, RAB27B could regulate the transition of the cell cycle from the G1 to the S phase, and the concept of whether RAB27B exerted this function through p21 was put forward. The protein level of p21^waf/Cip1^ was determined after cells were transfected with siR for 72 hours (Figure 6A). Western blot analysis showed that a depressed level of RAB27B resulted in elevated expression of p21^waf/Cip1^, indicating that RAB27B could regulate the expression of p21^waf/Cip1^.

To clarify potential mechanisms and regulatory networks, a PI3K/AKT pathway inhibitor (LY294002, 20 μm) was delivered to BEL7402 cells to identify expression involving their downstream pathways. The results revealed that the expression of p-PI3K and p-AKT(Ser473) were decreased and that of p21 was increased compared with the vehicle group after adding LY294002 for 24 hours (Figure 6A). These results suggest that RAB27B may affect cell cycle progression and proliferation of HCC cells through the PI3K/AKT pathway.

The opposite results were obtained from recovery experiments. Western blot assays showed an obvious increase in the levels of p-PI3K and p-AKT after transfection with RAB27B plasmids for 48 hours. With activation of the PI3K-AKT pathway, the expression of p21 was very considerably decreased (Figure 6A). As a secretory small GTPase, the levels of RAB27B had also been evaluated in the cell supernatant when the cells were treated by knockdown or overexpression. With the elevated or decreased changes of cell expression, the expression of RAB27B in the culture supernatant was up- or down-regulated accordingly (Supplementary Figure 2B). Grey values of p-PI3K, p-AKT and p21 were also analyzed to evaluate the quantity of protein, and levels in the siR/LY294002/RAB27B group were significantly changed compared to the SCR/vehicle/NC group (Figure 6B, 6C and 6D).

### RAB27B knockdown inhibits tumor growth *in vivo*

**Figure 7:**
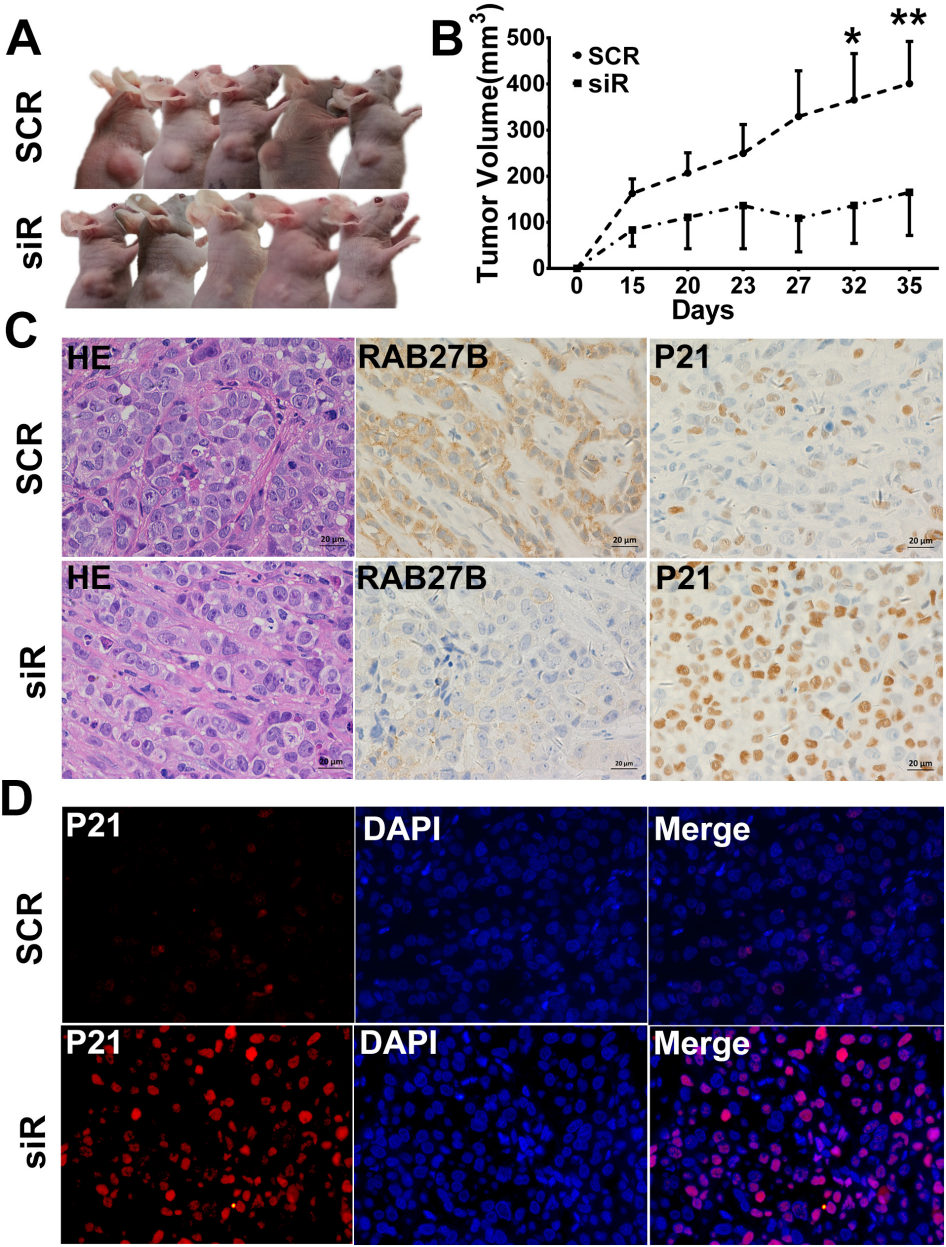
RAB27B downregulation inhibits tumor growth *in vivo* **(A, B)** The right upper sub-axillary of BALB/C nude mice was subcutaneously transplanted with BEL7402 cells transfected with GFP-SCR or siR plasmids (n = 6). Thirty-five days after implantation, RAB27B deficiency inhibited tumor growth in nude mice. The volume of the tumors was calculated and are presented as the mean±S.E.M. **(C)** H&E staining is shown in the left panel (400 × magnification). RAB27B and p21 expression in tumor tissues were detected by immunohistochemistry (400 × magnification). **(D)** Expression and intracellular localization of p21 was analyzed in tumor tissues by immunofluorescence (400 × magnification). *p < 0.05, **p < 0.01.

To further investigate the biological functions of RAB27B *in vivo*, a subcutaneous xenograft model was developed in nude mice. BEL7402 cells transfected with SCR and siR plasmids were harvested and injected into the armpit of nude mice at a concentration of 10^7^ cells. Tumor diameters were measured every 3 to 5 days. The results revealed that downregulated expression of RAB27B in BEL7402 cells significantly lead to diminished tumor sizes than those in the SCR group (Figure 7A and 7B). Additionally, the protein level of RAB27B in tumors was determined by IHC and IF analysis, the results of which confirmed that levels in the siR group were much lower than in the SCR group (Figure 7C). At the same time, p21^waf/Cip1^ expression was analyzed by IHC and IF assays. In the tumors from the siR group, the p21^waf/Cip1^ level was much higher than in those from the SCR group (Figure 7C and 7D). These results suggested that, *in vivo*, a low level of RAB27B inhibits HCC cell ectopic tumor formation and growth by maintaining the localization and stability of p21 to exert an inhibitory effect in tumor proliferation.

## DISCUSSION

Belonging to the secretory Rab27 subfamily, RAB27B has no significant expression in normal tissues, with the exception of cells that are often under mechanical traction pressure and several secretory cells, for example, certain brain cells [17]. Recent research has revealed that the expression of RAB27B is significantly increased in tumors. Increased expression of RAB27B is associated with malignant progression of ovarian cancer [21], glioblastomas [22], colorectal cancer [23] and HCC [20]. In estrogen receptor (ER)-positive breast cancer cell lines, RAB27B was found to promote cell growth, invasion, and metastasis by secreting pro-invasive signaling molecules, and its high expression was associated with poor prognosis [19]. Moreover, expression of RAB27B was upregulated in HCC tissues, and correlated with tumor progression and poor prognosis [20].

Based on the above findings, our study aimed to determine its value in early diagnosis and evaluate the mechanisms by which RAB27B plays a role in HCC progression. First, we chose HCC patient serum samples, since these are readily obtained from simple clinical procedures. Elevated expression of RAB27B in HCC patient sera was related to decreased OST and malignancy progression. Increased expression was also correlated with shorter OST both in BCLC BCD stages and TNM grades I-IV. K-M analysis revealed that the expression of RAB27B in serum of patients with BCLC stage A was not related to OST. While in BCLC stages BCD, patients with higher expression levels of RAB27B tended to exhibit a poorer prognosis. We suggest that these results show a correlation between serum RAB27B and HCC progression. For patients in BCLC stage A, their health was typically relatively normal and liver function was not unusual. Hence, for patients in BCLC stage A, correlation between RAB27B expression and prognosis was not prominent. Therefore, we believe that the increasingly significant role RAB27B played might not be evident in the early stages of disease development, but, rather, during the progression of HCC. Similar to the expression results identified in tissues, as a secretory small GTPase, RAB27B was significantly elevated in the serum and plasma of patients with hepatocellular carcinoma, but was lower in patients with chronic hepatitis B and in healthy individuals, indicating that it may be a potential biomarker for early diagnosis and as a therapeutic target in HCC.

Second, we studied the biological functions of RAB27B in HCC cells. Decreased expression of RAB27B could lead to suppression of cell growth both *in vitro* and *in vivo*. In cell culture supernatants, the expression of RAB27B protein also changed, corresponding to its intracellular expression levels. These results indicated that RAB27B might play an important role during the occurrence and development of HCC.

In eukaryotic cells, protein and signaling molecule transport mostly depends on vesicle trafficking, including endocytosis and exocytosis between different organelles as well as between cells and the extracellular microenvironment [14]. This activity is largely regulated by a series of highly-conserved proteins including Rab GTPases. Rab GTPases, which are intracellular transport proteins, modulate vesicular transport and act as molecular switches in response to the GTP/GDP cycle [16]. A series of studies indicated that vesicle trafficking and exocytosis are vital processes in tumorigenesis, and the Rab family may be an important part of these processes [24–26]. By means of transportation of cytokines, growth factors, proteins, and RNAs, the exchange of substances inside and outside the cell is accomplished. The high expression of EGFR and VEGF in tumors has been shown to be directly correlated to the expression of members of the Rab family. Rab7 was demonstrated to regulate late endocytosis with lysosomal fusion where EGFR is degraded. In glioblastomas, the high expression of both Rab7 and EGFR was shown in cell lines and xenografts [27]. RabEX-5, a guanine-nucleotide exchange factor (GEF) for Rab-5, plays a significant role in the development of gastric cancer by activating the vascular endothelial growth factor signaling (VEGFR) pathway [28].

RAB27B, a secreted Rab GTPase, modulates vesicle exocytosis intracellularly [29]. On knocking down the expression of RAB27B in HCC cells, we found that the PI3K/AKT pathway was inhibited. Accompanying this, both P-PI3K and p-AKT expression were decreased. AKT has been found to attach to the cytoplasmic membrane before activation. Once activated by growth factors, it is transferred to the nucleoplasm [30]. In HCC cells, with an increase in RAB27B expression, an elevation in extracellularly-secreted cytokines may stimulate the PI3K/AKT pathway, which can promote cell proliferation and inhibit cell apoptosis.

Furthermore, in this study, flow cytometry results revealed that suppression of RAB27B inhibits the G1/S transition. Accompanying these processes, the expression of p21, a CDKI, was remarkably increased after knockdown of RAB27B in BEL7402 cells and in transplanted subcutaneous tumors. To further clarify potential pathways, PI3K inhibitors (LY294002, 20 μm) were used to detect protein expression in the pathway. The results revealed that p-PI3K and p-AKT-(Ser473) expression was significantly decreased and p21 levels were greatly elevated after addition of LY294002 for 24 hours to BEL7402 cells. Studies have identified that the growth-inhibitory function of p21 is closely related to its intracellular localization [13, 31], whereas cytoplasmic re-localization resulting from phosphorylation of p21 was associated with anti-apoptotic behaviors [32]. P-AKT was able to phosphorylate p21 at T145, and transfer p21 to the cytoplasm, where it promotes cell growth and suppresses cell apoptosis [12, 33, 34].

Based on our findings, we hypothesize that increased expression of RAB27B may lead to increased vesicle exocytosis in HCC. High levels of growth factors might activate AKT, promoting translocation of p-AKT to the nucleus, where p21 is then phosphorylated, thus promoting cell growth and inhibition of apoptosis. In contrast, knock-down of RAB27B may suppress vesicle exocytosis, and phosphorylation of AKT was correspondingly diminished, according to our results. The inactivation of AKT stabilizes and upregulates p21 in the nucleus, resulting in the inhibition of cell growth and promotion of G1/S arrest.

However, one limitation of our study lies in the lack of pathological diagnosis of patients with HCC. In this work, we have identified that serum RAB27B might be a serum biomarker in HCC. However, according to some of the surrounding literature, RAB27B plays a significant role in the formation and secretion of platelet dense granules [35]. Moreover, it is also involved in neutrophil exocytosis [36]. To exclude interfering factors, we tried to use refrigerated centrifugation to separate blood samples and then detected RAB27B expression in the plasma. Ultimately, similar results were measured in plasma as well as in serum. However, due to the small number of cases, the results lack statistical robustness and baseline comparison. In combination with the above results, and considering the accuracy of serum manipulation, it is possible to consider whether RAB27B expression in serum or plasma exhibit positive correlations. Additionally, further study and statistical analysis are warranted in exploration of expression in plasma. Moreover, elucidation of the mechanisms underlying RAB27B function in cellular pathways through exocytosis is worth further research and investigation.

In summary, our study revealed that serum RAB27B expression was remarkably elevated in patients with HCC, compared to healthy controls and patients with chronic hepatitis B infection. In patients with HCC, higher serum levels of RAB27B were associated with poorer prognosis and shorter OSTs. Knockdown of RAB27B may inhibit the PI3K/AKT pathway, suppressing cell proliferation, and upregulation and stabilization of p21, thereby leading to cell cycle arrest. This is the first study to reveal the mechanism by which RAB27B promotes cell proliferation via the PI3K/AKT/p21 signaling pathway. Serum expression of RAB27B, as a more convenient detection method, could be a potential diagnostic and therapeutic target, having clinical significance in predicting HCC progression and prognosis.

## MATERIALS AND METHODS

### Patients and clinical samples

Serum samples were individually collected from 154 patients with HCC, 40 normal controls, and 31 patients with chronic hepatitis B infection and were obtained from the Beijing Ditan Hospital affiliated to Capital Medical University. Patients with HCC selected in the randomized controlled clinical study were diagnosed with hepatitis B virus (HBV)-HCC between 2014 and 2015, and their sera were collected and tested as baselines. They subsequently received standardized treatment for HCC [37]. Serum samples from normal individuals were collected from outpatients who underwent health examinations in 2016. This study was approved by the institutional ethics committee of Beijing Ditan Hospital (Beijing, China). Written informed consent was given by all participants. Patients’ corresponding clinical materials are summarized in Table 1.

### Cell lines, cell infection and transfection

HCC cell lines (BEL7402, SMMC, MHCC97H, MHCC97L, and HuH-7) were purchased from the China Infrastructure of Cell Line Resources. Cells were cultured in Dulbecco’s modified Eagle’s medium (DMEM) supplemented with 10% fetal bovine serum (FBS), 1% penicillin/streptomycin, and 1% glutamine. All cells were incubated in a 37°C atmosphere supplemented with 5% CO_2_, and passaged once every 2-3 days. The siRNA-RAB27B (siR) and scramble (SCR) lentiviruses containing the gene encoding green fluorescent protein (GFP) were packaged by Gene Pharma Inc. (Shanghai, China). BEl7402 cells were cultured at 8 × 10^4^ cells per well in 6-well plates and infected with siR- RAB27B and SCR lentiviruses, according to the manufacturer’s instructions. PI3K inhibitor LY294002 (sigma, 20 μm) were also added to BEL7402 cells for 24 hours. RAB27B plasmids, containing the complete open reading frame (ORF), were purchased from OriGene (OriGene Technologies, Rockville, MD, USA). Huh-7 cells were added at 10^5^ cells per well in 6-well plates and transfected with RAB27B and Negative Control (NC) plasmids using Lipo3000 (Invitrogen, Carlsbad, CA, USA) according to the manufacturer’s instructions.

### Real-time quantitative PCR

Total RNA was isolated from the normal liver cell line (MIHA), HCC cell lines (BEL7402, SMMC, MHCC97H, MHCC97L, and HuH-7), and transfected BEL7402 cells using TRIzol reagent (Invitrogen, USA) for analyses. RNA was reverse transcribed using the One Taq RT-PCR kit (New England Biolabs, USA). Quantitative reverse transcriptase polymerase chain reaction (RT-qPCR) analysis was performed using SuperReal qPCR PreMix (SYBR Green) (TIANGEN, CHINA) in a C1000TM Thermal Cycler and a CFX96TM Real-Time system (BIO-RAD, USA) according to the manufacturers’ protocols. To evaluate *RAB27B* expression, primers were designed as follows F: ATAAGTAGCTGTCCCCGTGC, R: TCAGCCTGCGAAGTTTCCTT, and were evaluated in comparison to the housekeeping gene *GAPDH*.

### Cell proliferation assays

MTT and colony formation assays were carried out to measure relative cell growth and colony formation capacity. After transfection with lenti-siRNA, lenti- SCR or NC/RAB27B plasmids for 24 hours, BEL7402 and HuH-7 cells were seeded at 2000 cells per well, where each group had five replicate wells in 96-well plates. In order to determine relative cell growth, the MTT assays were carried out for five days. Briefly, 20 μl of MTT solution (5 mg/mL) was added to each well and incubated at 37°C for 4 hours. The optical density at 490 nm was determined and the absorbance values for cells solubilized in 150 μl DMSO (Sigma) were used to approximate the number of live cells.

Colony formation assays were performed with 1000 cells plated in six-well plates for each group, and cells were infected for 24 hours. After 14 days of incubation, each well was washed with PBS and stained with crystal violet. All colonies were manually counted using a microscope (Leica DM6000 B; Upright Microscopes, Wetzlar, Germany).

### Cell migration and invasion assay

For the trans-well chamber assays, a filter membrane with an 8 μm pore membrane pre-coated with Matrigel (BD Biosciences, CA, USA) was used in invasion assays, while for cell migration assays ECM was omitted (Costar, NY, USA). BEL7402 and HuH-7 cells were cultured at a density of 1 × 10^4^ per upper well in 200 μl medium (DMEM, without FBS). 500 μl of complete medium (DMEM, 10% FBS) was added to the lower chamber. After incubation at 37°C for 24 hours, the membranes were fixed in 100% alcohol and stained with 1% crystal violet for 30 minutes separately. At least six random microscopic fields were counted by microscope.

### Western blot assay

Western blot assays were carried out to assess protein expression in lenti-SCR /siR plasmid-infected cells or NC/ RAB27B plasmid-transfected cells. Total protein was isolated in lysis buffer. After quantification of protein, equal amounts (40 μg) were added to sample wells, separated in 15% SDS-polyacrylamide gels, and transferred to polyvinylidene difluoride membranes. Western blotting analysis was performed with anti-rabbit antibodies against RAB27B (Proteintech, USA, 1:1500), P21^waf/Cip1^ (CST, USA, 1:1000), p-PI3K (CST, 1:500), AKT (CST, 1:1000), and p-AKT (Ser473, CST, 1:500). Anti-β-actin antibodies (Proteintech, 1:1000) and GAPDH antibodies (CST, 1:1000) were used to ensure equivalence of protein samples loaded onto the gel. RAB27B fusion protein (HIS tag, Proteintech, 200 pg/mL) was selected as a positive control. Alpha View software (ProteinSimple, USA) was employed to quantify the integrated density of the bands.

### Cell cycle analysis

BEL7402 cells infected with lenti- SCR /siR plasmids and HuH-7 cells transfected with NC/ RAB27B plasmids were plated in 6-well plates. After a 48-hour incubation, the cells were separately collected and fixed for 24 hours in 70% alcohol, and stained with propidium iodide for 30 min in the dark in a water bath at 37°C according to the manufacturer’s instructions (BD, USA). Subsequently, the cells were collected, and the cell cycle was analyzed using a flow cytometer (BD).

### Subcutaneous xenograft nude mouse models and treatment

Nude mice (BALB/c-A, male, 4 weeks old) were purchased from the animal center at Capital Medical University and kept in specific pathogen-free environments. Twelve mice were randomly divided into two groups, and the subcutaneous HCC tumor model of nude mice was established. After transfection with luciferase lentivirus, BEL7402 cells with stably-decreased expression of RAB27B or the SCR were planted subcutaneously into the armpit of nude mice (1 × 10^7^ cells). The sizes of tumor tissues were measured every 3 to 5 days for 35 days with a Vernier caliper. Mice bearing subcutaneous tumors were euthanized after 35 days. The tumor tissues were surgically resected, fixed in formalin and embedded in paraffin. Paraffin-embedded tissues were used for immunohistochemistry and immunofluorescence analysis.

### Immunohistochemistry and immunofluorescence

Formalin-fixed, paraffin-embedded tissue sections were de-paraffinized with a graded series of alcohol washes, followed by antigen retrieval and blockage with 5% BSA for 60 minutes. Tissue sections were incubated with antibodies against RAB27B (1:100 dilution, Proteintech) and p21^waf/Cip1^ (1:50 dilution, CST) for 60 minutes. For immunohistochemical staining, the procedures were conducted automatically by Biotin-Streptavidin HRP Detection System (ZSGB BIO, China). For immunofluorescence, tissue sections were incubated with antibodies against DAPI (1:100 dilution, ZSGB BIO, China) and p21^waf/Cip1^ (1:50 dilution, CST). Images were obtained using a ZEISS microscope (Carl Zeiss AG, Baden-Württemberg, Germany).

### Statistical analysis

Statistical analysis was performed using SPSS20.0 (IBM, NY, USA) and presented using GraphPad software (GraphPad Software, CA, USA). Categorical data were presented by the number of the observations. Variables conforming to normal distributions were presented as the mean±standard deviation. Non-normal distribution variables are indicated with the median (Q1, Q3). The relationship between OST and clinical variables was analyzed by univariate and multivariate analysis using a Cox proportional hazards model. Multivariate analysis was used to assess correlations between univariate meaningful variables and OST. Based on the above analysis, a receiver operating characteristic (ROC) curve including RAB27B expression and OST was constructed, and the area under the ROC curve (AUC) was calculated to evaluate its discriminatory capability. The cut off value was calculated as its maximum sensitivity and specificity of RAB27B expression, and patients were divided into high- RAB27B expression (high expression) and low- RAB27B expression (low expression) groups based on the cut off value. K-M survival curves were constructed to compare OSTs between these two groups, and the differential analyses were performed by log rank test. All experiments were repeated three times. Differences in RNA expression of RAB27B, tumor cell proliferation, colony formation number, and cell numbers in the various stages of cell cycle between the experimental groups were analyzed by Student’s t-test. A *p*-value of < 0.05 was considered statistically significant.

## SUPPLEMENTARY MATERIALS FIGURES


