# Supplementary material for: Downregulation of serum RAB27B confers improved prognosis and is associated with hepatocellular carcinoma progression through PI3K-AKT-P21 signaling
Source: Oncotarget. 2017 May 19;8(37):61118–32. doi: 10.18632/oncotarget.18010 (PMC5617411; doi:10.18632/oncotarget.18010)
Supplement: Supplementary file 1 [file oncotarget-08-61118-s001.pdf]

Downregulation of serum RAB27B confers improved prognosis and is associated with hepatocellular carcinoma progression through PI3K-AKT-P21 signaling

SUPPLEMENTARY MATERIALS

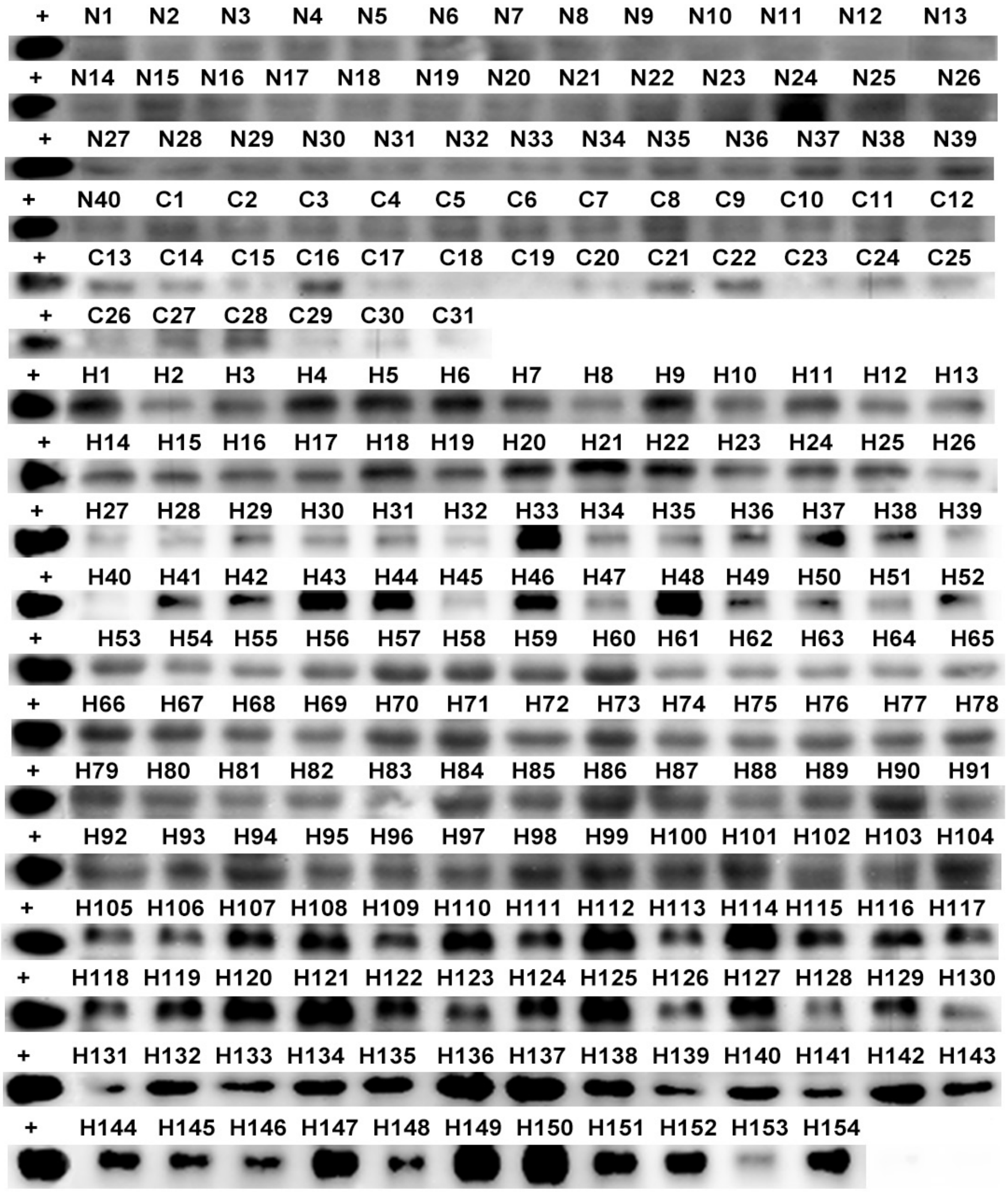

Supplementary Figure 1: Serum expression of RAB27B in normal human, patients with chronic hepatitis B infection, and patients with hepatocellular carcinoma. N = NOR = normal humans, C = CHB = chronic hepatitis B, H = HCC = hepatocellular carcinoma.

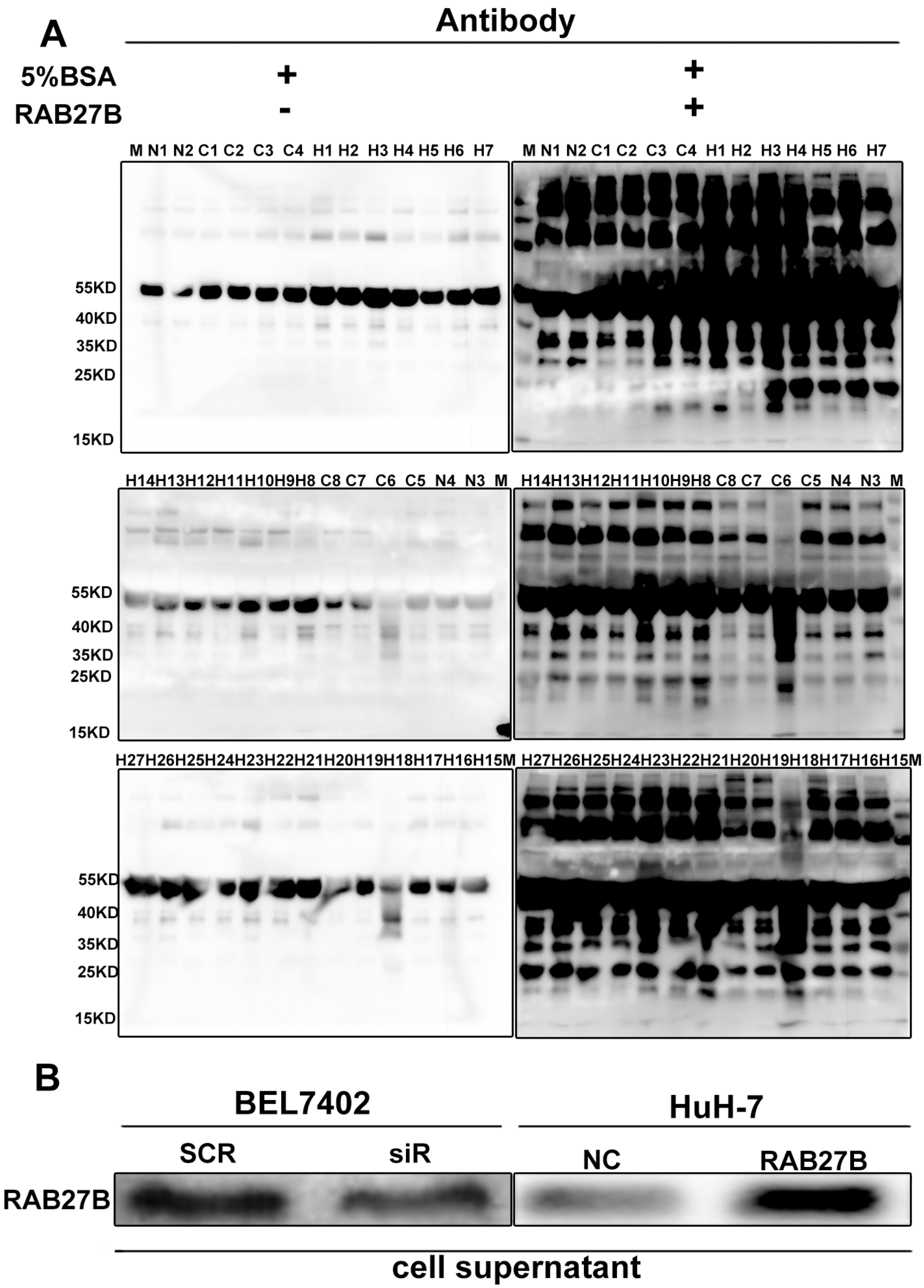

Supplementary Figure 2: Serum expression of RAB27B with intact membranes. (A) RAB27B expression in serum with intact membranes with or without primary antibody. **(B)** The expression of RAB27B in the supernatant of knockdown or over-expressing cells. N = NOR = normal individuals, C = CHB = chronic hepatitis B, H = HCC = hepatocellular carcinoma, M = marker.
